# Commensal and Opportunistic Bacteria Present in the Microbiota in Atlantic Cod (*Gadus morhua*) Larvae Differentially Alter the Hosts’ Innate Immune Responses

**DOI:** 10.3390/microorganisms10010024

**Published:** 2021-12-24

**Authors:** Ragnhild Inderberg Vestrum, Torunn Forberg, Birgit Luef, Ingrid Bakke, Per Winge, Yngvar Olsen, Olav Vadstein

**Affiliations:** 1 Department of Biotechnology and Food Science, Norwegian University of Science and Technology, 7491 Trondheim, Norway; ragnhild@morefish.no (R.I.V.); torfo@biomar.com (T.F.); b.luef@biology.leidenuniv.nl (B.L.); Ingrid.bakke@ntnu.no (I.B.); 2 Department of Biology, Norwegian University of Science and Technology, 7491 Trondheim, Norway; per.winge@ntnu.no (P.W.); yngvar.olsen@ntnu.no (Y.O.)

**Keywords:** Atlantic cod, microbiota, innate immune system, germ-free, gnotobiotic

## Abstract

The roles of host-associated bacteria have gained attention lately, and we now recognise that the microbiota is essential in processes such as digestion, development of the immune system and gut function. In this study, Atlantic cod larvae were reared under germ-free, gnotobiotic and conventional conditions. Water and fish microbiota were characterised by 16S rRNA gene analyses. The cod larvae’s transcriptional responses to the different microbial conditions were analysed by a custom Agilent 44 k oligo microarray. Gut development was assessed by transmission electron microscopy (TEM). Water and fish microbiota differed significantly in the conventional treatment and were dominated by different fast-growing bacteria. Our study indicates that components of the innate immune system of cod larvae are downregulated by the presence of non-pathogenic bacteria, and thus may be turned on by default in the early larval stages. We see indications of decreased nutrient uptake in the absence of bacteria. The bacteria also influence the gut morphology, reflected in shorter microvilli with higher density in the conventional larvae than in the germ-free larvae. The fact that the microbiota alters innate immune responses and gut morphology demonstrates its important role in marine larval development.

## 1. Introduction

The roles of the microbiota associated with vertebrate hosts, including fish, have received much attention over the last decade. Several studies have shown that the microbiota stimulates the immune system and functions as a barrier against potential pathogens [1,2,3,4], aids in epithelial development and maturation [4,5] and affects the digestion of nutrients [6,7]. There is a bias in the type of animals studied, and still relatively few studies are published on the function of microbiota in fish. Most of the bacteria associated with the fish are harmless or beneficial [8,9]. However, specific pathogens and opportunistic bacteria are also present [10,11], and bacteria present in the natural environment of the fish cause many of the infections that are associated with the mortality of marine fish larvae [12].

Germ-free animals have been popular tools used in studies of host–microbe interactions [13,14], and the use of gnotobiotic zebrafish is well-known [15,16]. Rawls et al. [16] observed that gut microbiota in zebrafish stimulated proliferation of intestinal epithelial cells, as previously seen for rodents [5,17]. They found 212 genes that were differentially regulated in germ-free fish compared to fish exposed to bacteria. Moreover, 59 of those gene-expression responses were observed in both mice and zebrafish. These genes are involved in epithelial proliferation, nutrient metabolism and innate immune responses [16]. Our group have developed a protocol and a cultivation system for germ-free and gnotobiotic Atlantic cod larvae [18]. Using this system, Forberg et al. [19,20] showed that bacteria regulate the transcription of genes involved in immune response and nutrient digestion in the cod larvae, and that the response differs depending on whether the bacteria are dead or alive.

However, at that time, molecular tools for Atlantic cod were poorly developed, and this allowed characterisation of only a limited part of the host transcriptome. Following the sequencing of the Atlantic cod genome [21], it was discovered that cod lack certain families of cell surface receptors, whereas others are expanded, compared with zebrafish and mice [21,22,23,24]. The functional consequences of this evolutionary divergence in immune response with regards to host–microbe interactions have, to our knowledge, not been studied. Thus, the knowledge about the early regulation of the immune system of cod and its responses to both pathogenic and non-pathogenic bacteria is still limited.

The aim of this study was to increase our knowledge about host responses induced by the early microbial colonisation of Atlantic cod larvae, focusing on the roles of microbes in regulation of the immune system and digestion. Newly hatched cod larvae were reared under three different conditions: germ-free, gnotobiotic (two probiotic candidate strains added) and conventional (uncontrolled microbial environments). The water- and fish-associated microbiota was characterised and analysed, and host responses were examined using a custom oligo microarray as well as transmission electron microscopy (TEM).

## 2. Materials and Methods

The experiment was carried out within the Norwegian animal welfare act guidelines, in accordance with the Animal Welfare Act of 20 December 1974, amended 19 June 2009, at a facility with permission to conduct experiments on fish (code 93) provided by the Norwegian Animal Research Authority (NARA). The experiment was approved by NARA.

### 2.1. Cod Larval Rearing

Atlantic cod eggs were delivered from Nofima Marin national breeding station (Havbruksstasjonen Tromsø, Norway). Upon arrival, the cod eggs (55–65-day degrees) were acclimatised in filtered (0.22 µm Micropore^®^) autoclaved (121 °C, 20 min) seawater (FASW) at 6 ± 1 °C, in the dark. Germ-free larvae were obtained according to the protocol of Forberg et al. [18] (information about germ-free verification in Text S1). Cod larvae (65 larvae in 2 L water) were reared under three different conditions: germ-free, conventional and gnotobiotic. For the gnotobiotic treatment, two different bacterial strains were added in equal amounts (final density of 10^6^ cells/mL) to the rearing bottles: *Microbacterium* ND 2–7 and *Vibrio* RD 5–30, both previously isolated from cod and identified as probiotic candidates (for details, see [25]) (information about live feed and bacterial cultures in Text S2). The rearing bottles representing the conventional condition were filled with microbially matured water, from a biofilter in a seawater lab-scale aquaculture system. After stocking, the temperature was increased by 1 °C/day until 12 °C was reached.

### 2.2. Sampling

Each treatment had 11 replicate bottles at trial start. Cod larvae were collected at 4 (only for DNA extraction), 8, 13 and 16 dph. Fish from one bottle were sampled at 4 dph, while 3 replicate bottles were sampled at 8, 13 and 16 dph. Water was sampled from one bottle at 1 and 4 dph, and three replicate bottles at 8, 13 and 16 dph. After sampling, the bottles were taken out of the experiment, thus reducing the number of replicate bottles with time. Larvae were sacrificed with an overdose of tricaine methanesulfonate (MS-222) prior to sampling, snap-frozen in liquid nitrogen and stored at −80 °C for further analyses. To investigate larval growth, 10 individual larvae from each sampled bottle were freeze-dried and weighed. More details regarding sampling procedures and DNA/RNA extraction are described in Supplementary Materials (Text S3).

### 2.3. Characterisation of Microbial Communities

The water and fish samples from the gnotobiotic treatment were analysed by DGGE. PCR products representing the V3 region of the 16 S rRNA gene were generated using a nested PCR protocol to avoid possible amplification of eukaryotic 18S rDNA [26]. The PCR was set up and analysed as described by Bakke et al. [27].

For in-depth analysis of the microbiota in the conventional treatment, Illumina MiSeq sequencing was performed based on total DNA extracted from water and larvae sampled at 1 (only water), 4, 8, 13 and 16 dph. Larval and water samples were prepared for Illumina MiSeq sequencing by amplification of the V4 region of the 16S rRNA gene, by using the following primers (bacteria-specific V4 primer, underlined and bold) including 5′ overhang, as suggested by Illumina:

515F F′ TCGTCGGCAGCGTCAGATGTGTATAAGAGACAGNNNN**GTGCCAGCMGCCGCGGTAA** 3′ and

803 R 5′ GTCTCGTGGGCTCGGAGATGTGTATAAGAGACAGNNNN**CTACVVGGGTATCTAAKCCBK** 3′.

The amplicon library preparation and processing of the Illumina sequencing data was performed as described by Vestrum et al. [28]. In short, for the first stage of amplification, the reactions were run for 38 cycles for water samples and 40 cycles for cod larval samples (98 °C 15 s, 55 °C 20 s, 72 °C 20 s), with 0.3 µM of each primer, 0.25 mM of each dNTP, 2 mM of MgCl_2_, 12 µM of BSA, glycerol (10%), Phusion Hot Start II High-Fidelity DNA Polymerase and reaction buffer from Thermo Scientific in a total volume of 20 µL. All samples were normalised using the SequalPrepTM Normalisation Plate Kit (Invitrogen). A second PCR was performed to attach dual indices and Illumina sequencing adapters to the normalised v4 amplicons by using the Nextera XT Index Kit. The indexed PCR products were normalised as described above, pooled, and concentrated by using Amicon^®^ Ultra-0.5 Centrifugal Filter Devices. The resulting amplicon library was sequenced on a MiSeq lane (Illumina, San Diego, CA, USA) with v4 reagents employing 260 bp paired-end reads at the Norwegian Sequencing Center at the University of Oslo, Norway. The Illumina sequencing data were processed with the high-performance USEARCH utility (version 11) (http://drive5.com/usearch/features.html (accessed on 22 March 2019). Taxonomy assignment was performed applying the Sintax script [29] with a confidence value threshold of 0.8 and the RDP reference dataset (version 16). OTUs of particular interest were further analysed with the RDP tools [30] Classifier and Sequence Match. OTUs representing algae, Archaea and Cyanobacteria/Chloroplast were removed from the OTU table. In addition, an OTU representing Propionibacterium acne, a well-known contaminant of DNA extraction kits [31], was removed. To remove biases due to variation in sequencing depth, analyses were performed on an OTU table that had been subsampled to 15500 sequencing reads for each sample. The subsampling threshold was chosen based on the sample with the lowest number of reads in order to keep all samples in the dataset and was performed to avoid bias due to differences in sequencing depth. The resulting Illumina sequencing data were deposited at the European Nucleotide Archive (accession numbers ERS8484975-ERS8484994).

### 2.4. Microarray Design, Hybridisation and Annotation

A custom, Agilent 44 k oligo microarray (A-MEXP-2226, ArrayExpress, EMBL-EBI) described by Kleppe et al. [32] was used and analysed as described by Vestrum et al. [33]. This microarray design is partly based on the Atlantic cod gene set described by Star et al. [21] as well as EST sequences from various cod tissues/developmental stages. The identified differentially regulated transcripts were used for biological term enrichment analysis and Gene Ontology term (GO term) annotation in DAVID (Database of Annotation, Visualisation and Integrated Discovery) [34,35] (using the official gene symbol for human homologues).

### 2.5. Electron Microscopy Procedures

For processing fish larvae for transmission electron microscopy (TEM), the protocol from Galloway et al. [36] was adopted. Shortly, germ-free and conventional reared fish larvae from 16 dph were fixed in a mixture of 2.5% paraformaldehyde, 2.5% glutardialdehyde, 0.5% sucrose and 0.11 M HEPES buffer (pH 7.4) and stored at 4 °C until further processing. Three larvae from each treatment were rinsed in 0.11 M HEPES buffer, post-fixed in 2% OsO4 in 1.5% potassium ferricyanide (final concentration), bulk contrasted in 1.5% uranyl acetate and dehydrated in ethanol before embedding in Epon. After polymerisation, 50–60 nm sections of the fish midgut were cut using a Leica UC6 Ultramicrotome. These ultrathin sections were collected on 200-mesh copper grids and contrasted with 4% uranyl acetate and 1% lead citrate. Sections were inspected with a FEI Company Tecnai 12 operated at 80 kV and imaged using a digital MORADA G3 CCD camera (EMSIS).

### 2.6. Intestinal Morphometry and Statistical Methods

Computerised morphometric measurements of microvilli lengths (from tip to base, l), diameter (2r) and abundance of microvilli (µm^–2^) in the midgut of the fish were made using the image processing program iTEM (Olympus Soft Imaging Solutions GmbH). Microvilli parameters were measured according to the criteria of Brown [37]. Three fish larvae per treatment were investigated (*n* = 3). For length measurements, at least 65 microvilli per fish were analysed; in total, 318 microvilli within the germ-free and 294 microvilli within the conventional treatment were measured. For diameter measurements, at least 58 microvilli per fish were analysed; in total, 214 microvilli within the germ-free and 287 microvilli within the conventional treatment were measured. To quantify the abundance of microvilli, microvilli within a total area of around 400 µm^2^ were counted. iTEM was used to adjust contrast in the images and to insert calibrated scale bars into images.

### 2.7. Statistical Analyses

Student’s *t*-test (unpaired) was used to investigate significance in differences in Shannon indices, abundance of individual DGGE bands and larval growth measurements. Survival analysis was performed by the Kaplan–Meier method, and the Log-rank test was used for pairwise post hoc comparisons of survival across the groups. Ordination by Principal Coordinate Analysis (PCoA) based on Bray–Curtis similarities was used to visualize differences between sample groups, and one-way and two-way PERMANOVA based on Bray–Curtis similarities were used to test for statistically significant differences between sample groups. Similarity Percentage analysis (SIMPER) was used to identify OTUs responsible for differences (measured as Bray–Curtis similarities) between different sample groups. The multivariate analyses were performed using the program package PAST version 3.22 [38]. Venn diagrams were created using jvenn [39]. The Usearch commands Alpha_div and Sintax_summary were used to calculate alpha diversity indices and to generate taxa summary tables (at various taxonomic levels, as specified with the results), respectively.

Data from the intestinal morphometric study were statistically analysed with IBM SPSS Statistics (SPSS for Windows, version 26.0; SPSS Inc., Chicago, IL, USA). A Welch test was performed to investigate significant differences between the axenic- and conventional-treated fish regarding microvilli length, diameter and abundance of microvilli (µm^–2^). Differences were considered statistically significant when *p* ≤ 0.05.

## 3. Results

### 3.1. Larval Survival and Growth

Daily counts of dead larvae in the rearing bottles were used to calculate the percent of survival. Kaplan–Meier survival curves showed a clear separation of the conventional group vs. the germ-free and gnotobiotic group cumulative mortality, and this difference was highly significant (Log rank post hoc *p*-values 0.000032 and 0.000005 for the pairwise comparisons) (Supplementary Figure S1) (84.9%, 84.8% and 76.0% survival, respectively). At 16 dph, the dry weight of germ-free larvae was significantly lower (average 75.5 µg) than the gnotobiotic and conventional larvae (average 96.9 and 110.1 µg, respectively) (*p* = 0.017 and 0.059, respectively) (Supplementary Figure S2).

### 3.2. Composition of Fish and Water Microbiota

The DGGE profiles for the samples from the gnotobiotic rearing bottles (Supplementary Figure S3), where only two bacterial strains were added, were consistently identical, except for the presence of one additional band in one fish sample at 8 dph. This suggests the presence of a contaminating bacterial strain. Even though bacteria were added to the same final cell density in the rearing water in the gnotobiotic treatment, *V. gallicus* clearly dominated both in water and fish samples. *Microbacterium* was detectable at low levels in water samples and present only in some fish samples.

**Figure 1:**
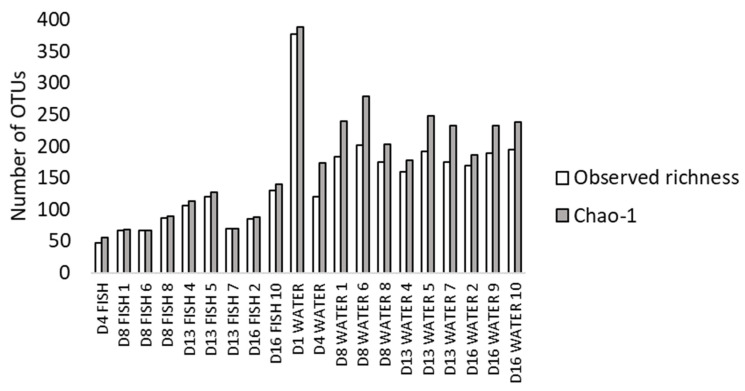
Observed richness (number of OTUs) and Chao1 values in fish and water microbiota at 1 (only water) to 16 dph (D1–D16). Numbers indicate the rearing bottle sampled. One fish sample consisted of 5 pooled larvae from one rearing bottle.

Fish and water microbiota in the conventional treatment were characterised by amplicon sequencing. After quality trimming and chimera removal, 1,039,322 reads were obtained. Two water samples and two fish samples from 4 dph were removed due to low number of reads. The estimated total (Chao1) and observed number of OTUs for each sample (Figure 1) indicate a sequencing depth of on average 95% and 82% in fish and water samples, respectively. The observed richness was generally higher in water than in fish.

**Figure 2:**
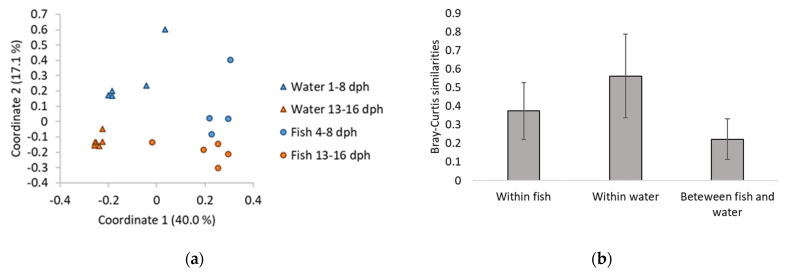
(**a**) PCoA ordination plot based on Bray-Curtis similarities for comparison of water and fish microbiota from samples taken early (1/4–8 dph) and late (13–16 dph) in the experiment. One fish sample consists of five pooled cod larvae from one rearing bottle. (**b**) Bray-Curtis similarities within fish and water microbiota, and between fish and water microbiota. Bars indicate standard deviation.

A PCoA ordination of the microbial communities in fish and water samples (Figure 2a) showed that the water and fish clustered separately. This was corroborated by Bray–Curtis similarities (Figure 2b). The PCoA plot also indicated that both the water and fish microbiota changed over time. There were significant differences between the water and fish microbiota both early (1/4–8 dph) and late (13–16 dph) in the experiment (one-way PERMANOVA, *p* = 0.04 and 0.01 for early and late, respectively) and also between water samples early and late in the experiment (one-way PERMANOVA, *p* = 0.01). There were no significant differences in the microbiota of fish samples early and late in the experiment.

**Figure 3:**
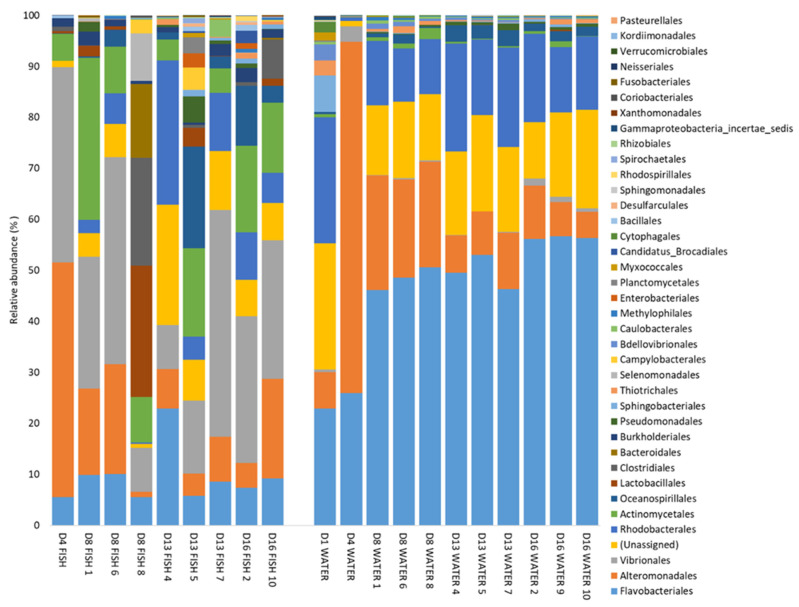
Taxa summary of bacterial orders detected at a relative abundance higher than 0.1% in at least one sample, at 1 to 16 dph (D1–D16). Numbers indicate the rearing bottle sampled. One fish sample consists of five pooled larvae from one rearing bottle.

The results from PERMANOVA analysis are reflected in the taxonomic composition of the microbial communities at the order level (Figure 3). The relative abundance of Vibrionales was more than 14 times higher in fish (up to 44%) than in water (≤3%). The relative abundances of Flavobacteriales in the water increased with time.

OTU 1 (*Polaribacter*, Flavobacteriales order) and OTU 2 (*Vibrio*) were the most abundant OTUs in the dataset. Both OTUs were present in both fish and water samples, but OTU 1 was far more abundant in the water than in the fish (on average 31% and 4% of the reads, respectively). OTU 2 had a higher relative abundance in the fish than in the water (average 21% and 0.4%, respectively). The third most abundant OTU was OTU 4 (*Colwellia*), which was more abundant early in the experiment (1–8 dph), both for fish (average 9.2%) and water (average 14.2%) samples, than later in the experiment (13–16 dph) (average 1.1% and 2.8% in fish and water samples, respectively).

### 3.3. Gene Expression in Atlantic Cod Larvae

At 8 dph, no genes in the cod larvae were differentially expressed between the treatments. For 13 dph samples, there were still no differentially expressed genes between conventional and gnotobiotic larvae. However, the genes *G-protein-coupled receptor family C group 6 member A* (*gprc6a*) (involved in regulation of inflammation, metabolism and endocrine functions) and *rhamnose binding lectin* (*rbl*) (involved in innate immunity) were downregulated in both conventional and gnotobiotic larvae compared with germ-free larvae. The *zg16* and *zg16-like* genes (involved in innate immunity) were also downregulated in gnotobiotic larvae compared with germ-free larvae. Only one gene, *lect2*, was upregulated in conventional larvae compared with germ-free larvae.

**Figure 4:**
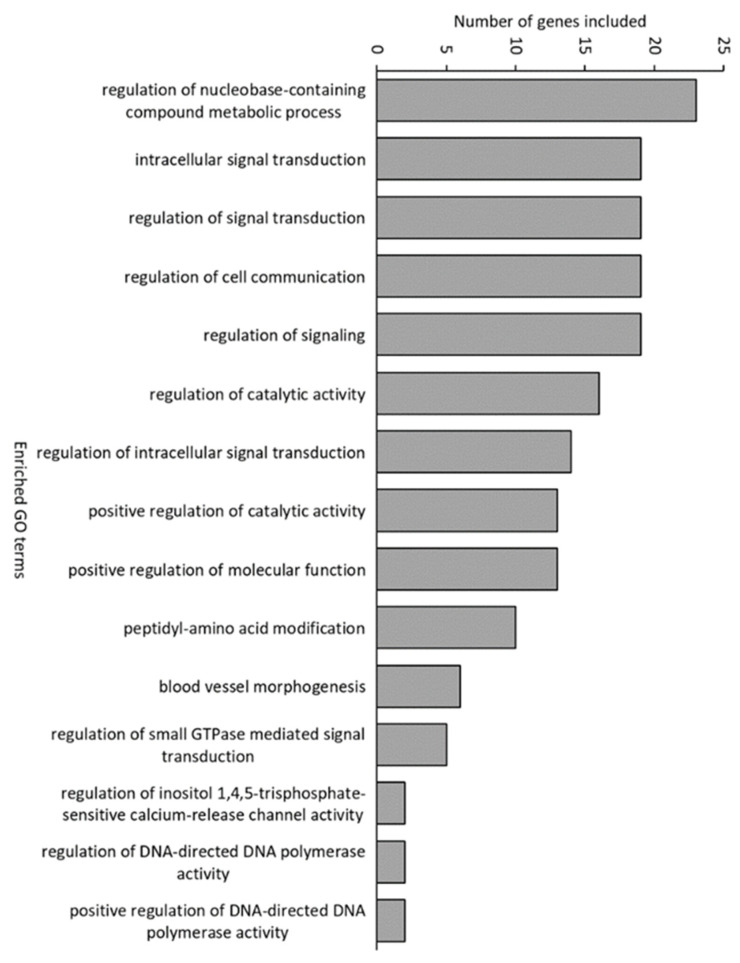
Significantly enriched Gene Ontology (GO) terms in conventional fish compared with germ-free fish.

For samples from 16 dph, 82 genes were downregulated and 97 were upregulated in conventional larvae compared with germ-free larvae. In gnotobiotic larvae, only 23 were downregulated and none were upregulated compared with germ-free larvae (Supplementary Tables S1–S3). Gnotobiotic and germ-free larvae generally showed similar expression profiles. Of the genes that were upregulated in conventional compared with germ-free larvae, 74% were also upregulated compared with gnotobiotic larvae (Supplementary Figure S4). Many of the genes that were upregulated in conventional compared with germ-free larvae are involved in innate immune responses and linked to signalling and glucose transport. Examples for immunity are, e.g., *interleukin 8* (*cxcl8*), *leukocyte cell-derived chemotaxin 2* (*lect2*) and *interleukin-1 receptor-activated kinase* (*irak1*), and for signalling/transport are, e.g., *solute carrier family 2 facilitated glucose transporter member 11-like* (*slc2a11*) and *solute carrier family 2 facilitated glucose transporter member 4-like* (*slc2a4*). Biological term enrichment analysis and Gene Ontology term (GO term) annotation in DAVID showed that 15 GO terms were enriched in conventional fish compared to germ-free fish (Figure 4). Several of the enriched GO terms, including the one with the highest number of genes (“regulation of nucleobase-containing metabolic process”), were related to growth and cell division. Other enriched GO terms were related to signalling and cell communication. Most of these GO terms were also enriched compared with gnotobiotic larvae (Supplementary Table S4). The KEGG pathway “bacterial invasion of epithelial cells” was enriched in conventional larvae compared with both gnotobiotic and germ-free fish.

However, 15 annotated genes had significantly lower expression in both conventional and gnotobiotic larvae compared with germ-free larvae. Interestingly, nine of these genes were involved in innate immune responses: *eosinophil peroxidase* (*epx*), *rbl*, *zymogen granule membrane protein 16* (*zgp16*), *myeloperoxidase precursor* (*mpo*), *Cytochrome b-245 heavy chain-like* (*cybb*), *immune-responsive gene 1 protein-like* (*Irg1*), *fish egg lectin* (*fel*), *N-acetylmuramoyl-L-alanine amidase-like* (*pglyrp1*) and *transmembrane protease serine 9-like* (*tmprss9*). Thus, the presence of bacteria, both as complex communities and simple gnotobiotic associations, downregulated some of the innate immune responses in the cod larvae.

**Figure 5:**
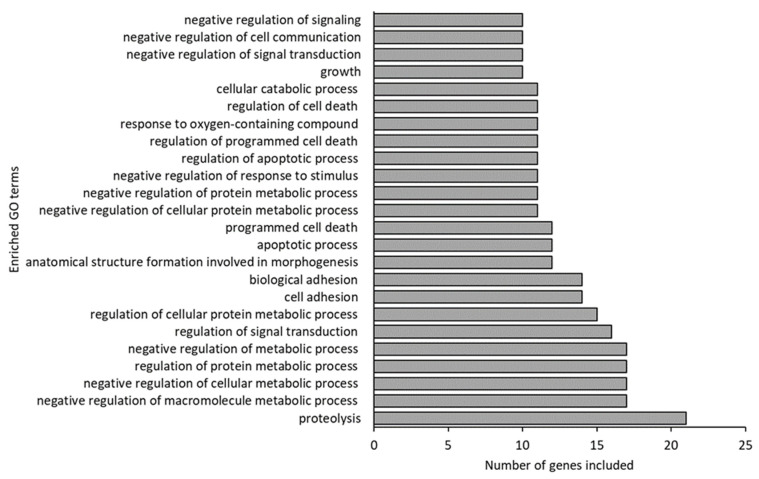
Enriched Gene Ontology (GO) terms (including 10 or more of the 52 input genes) in germ-free fish compared to conventional fish.

Far more GO terms differed between germ-free and conventional larvae than between germ-free and gnotobiotic larvae (95 and 11, respectively). The most enriched GO terms in the germ-free larvae were “proteolysis”, “negative regulation and regulation of metabolic processes”, “signal transduction” and “adhesion and cell death” (Figure 5).

Of the 21 genes included in the GO term “proteolysis”, 5 were involved in the KEGG pathway of “protein digestion and absorption”. This indicates that a large fraction of the “proteolysis” GO term is related to the cod larvae’s digestion.

### 3.4. Ultrastructure and Morphometric Analysis of the Intestinal Tissue

**Figure 6:**
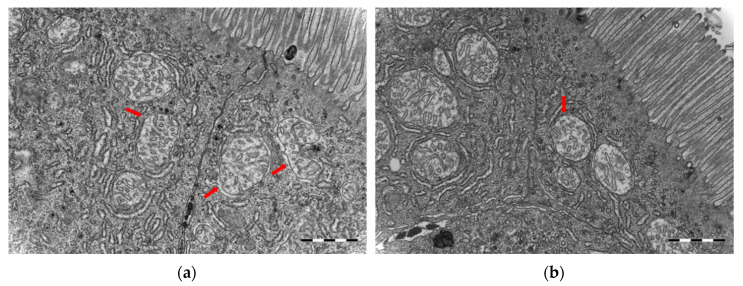
Transmission electron microscopy micrographs of mitochondria from the midgut of cod larvae at 16 dph reared under (**a**) germ-free and (**b**) conventional conditions. (**a**) Mitochondria in germ-free cod larvae were distorted, outer membrane showed discontinuities or was often missing and structures of Christae were reduced or hardly visible. (**b**) Mitochondria in conventional cod larvae showed clear Christae and a double membrane. Scale bars are 1 µm.

Comparison of the midgut ultrastructure, including tight junctions, microvilli disruption/damage, intercellular space and vacuoles, showed no significant differences between germ-free and conventional cod larvae. However, the mitochondria in germ-free cod larvae were distorted. The outer membrane showed discontinuities or was missing, and structures of the Christae were reduced or hardly visible (Figure 6). In contrast, the mitochondria in conventional cod larvae showed clear Christae and a clear double membrane.

**Figure 7:**
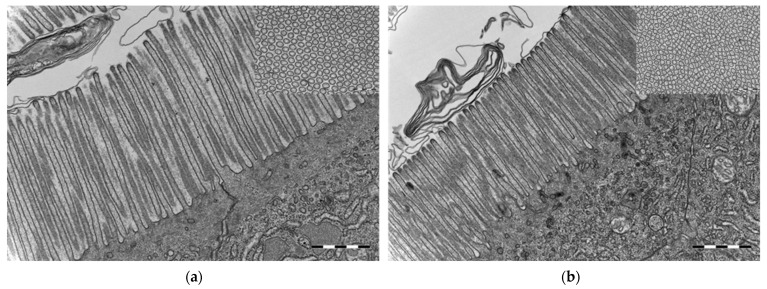
Transmission electron microscopy micrographs from the midgut of cod larvae at 16 dph reared under (**a**) germ-free and (**b**) conventional conditions. Microvilli in conventional cod larvae were shorter and thinner compared to the microvilli in germ-free cod larvae. Insets in (**a**,**b**): Transverse section of brush border—microvilli were closer to each other in the conventional than in the germ-free cod larvae. Scale bars are 1 µm and of insets 500 nm.

The microvillous brush borders in the midgut of cod larvae at 16 dph, reared under germ-free as well as conventional conditions, were well-defined and regular. Interestingly, germ-free cod larvae had significantly longer and significantly thicker microvilli than conventional cod larvae (for both analyses, Welch test, *p* ≤ 0.001, Figure 7). Moreover, the abundance of microvilli (µm^–2^) in the midgut was significantly lower in the germ-free larvae than in the conventional ones (Welch test, *p* ≤ 0.001, insets in Figure 7).

Microvilli were significantly closer to each other in the conventional than in the germ-free cod larvae. The morphometric measurements for the microvillous length, abundance and diameter are summarised in Table 1.

## 4. Discussion

In this study, gnotobiotic husbandry of Atlantic cod larvae was used as a tool to study host–microbe interactions. Cod larvae were reared under three different treatments: germ-free, gnotobiotic (*Vibrio gallicus* and *Microbacterium* added) and conventional. The only difference between the cod larvae in the conventional treatment and those in the gnotobiotic and germ-free treatments was that the microbial conditions in the conventional treatment were uncontrolled.

For the transcriptomic analysis, the major differences between the treatments were observed at 16 dph, and therefore the discussion is focused on this time point. At 16 dph, the gut of the larvae is larger and more developed, and thus the number of niches available for bacteria may be higher than at earlier life stages, and this may allow more bacteria to coexist through selection [40]. This was also reflected in our data, as the richness increased at this time point. Importantly, our analyses were based on pooled, homogenised whole fish, leading to conservative conclusions. Analyses at the organ level could possibly discover more differences in the gene expression patterns between the fish from the different treatments, also at earlier time points.

### 4.1. Microbial Environments

DGGE analyses of the microbiota from the gnotobiotic treatment showed that *V. gallicus* dominated both in water and fish samples, whereas *Microbacterium* was detectable at low levels in water samples but present in only some of the fish samples. This corroborated previous studies, where *V. gallicus* was found to adhere to and grow fast in mucus [25]. This could explain the higher abundance of *V. gallicus* compared to *Microbacterium* in the cod larvae in this experiment.

For the conventional treatment, we used 16S rDNA Illumina amplicon sequencing to characterise fish and water microbiota. The results show that the fish microbiota differed significantly from the water microbiota, corroborating earlier studies [33,41,42,43]. The water and larval microbiota were dominated by bacterial taxa considered to represent opportunistic, rapid-growing bacteria, such as Vibrionales, Actinomycetales, Alteromonadales and Flavobacteriales. This may be a result of r-selection in the water [44]. Pulses of organic matter originating from the addition of feed and fish defecation will create a high carrying capacity in the rearing bottles. When adding new water with lower carrying capacity to the rearing bottles, the microbe–microbe competition in the water is reduced, and this favoured growth of fast-growing, opportunistic species (r-strategists) [44]. Opportunistic bacteria could potentially be detrimental for the cod larvae, and this might be the reason for the lower survival observed in the conventional treatment than in the gnotobiotic and germ-free treatment. However, overall, the survival was good, and comparable to what is typically seen in first feeding experiments with cod larvae. In our dataset, 6 OTUs were classified as Vibrio. Using the Ribosomal database project (RDP) SeqMatch tool [30] to identify the most closely related type strains for each of them (Supplementary Table S5) showed that one OTU matched *V. campbellii* and one *V. anguillarum.* Both species are known fish pathogens [45].

Our results confirm that we had distinct microbial environments in the gnotobiotic and conventional treatments. The gnotobiotic treatment represents environmental conditions with non-detrimental host–microbe interactions. The conventional treatment, on the other hand, represents an environment characterised by the presence of opportunistic bacteria, which might have had detrimental effects on the fish. Thus, the conventional treatment does not represent a “natural” microbial environment, but rather a suboptimal microbial environment, including detrimental host–microbe interactions. This is reflected in the gene expression of the fish, and likely the reason why we see (1) increased expression of some genes related to inflammatory responses and oxidative stress, and (2) lower survival of conventionally reared fish than for the germ-free and gnotobiotic cod larvae.

### 4.2. Presence of Bacteria Downregulates Host Responses Related to Nutrient Utilisation and Innate Immune Responses

Members of the gut microbiota in other species are well-known to aid in the digestion of, e.g., complex carbohydrates [46] and proteins [47]. Since the gut of cod larvae is functionally immature at hatching [48,49], the enzymatic activity of bacteria may aid in digestion of the live feed organisms. Even if survival was very high for the germ-free cod, and the intake of feed appeared similar in all treatments, they gained less weight than the conventional larvae. The transcriptional responses in the germ-free larvae support that they had difficulties in digesting feed, as the most enriched GO term in germ-free larvae compared with conventional larvae was proteolysis. Of the 21 genes included in the GO term “proteolysis”, 5 were involved in the KEGG pathway of “protein digestion and absorption”. This indicates that a large fraction of the “proteolysis” GO term is related to the host’s digestion. Interestingly, germ-free rats also have higher activities of digestive enzymes, such as amylase and lipase, in their intestine than conventional rats [50]. Transcriptional responses related to fasting were downregulated in the conventional larvae compared with germ-free larvae. Host responses related to fasting were also found in the zebrafish study by Rawls et al. [16].

Similar to findings in germ-free rodents [51,52], the germ-free cod larvae showed significantly longer microvilli in the midgut than the conventional larvae (Figure 7, Table 1). This might be related to reduced renewal of the intestinal epithelium in these animals [52]. Similarly, albeit at a larger physiological scale, Willing et al. [53] found that germ-free pigs have longer villi and shorter crypts in their distal intestine, and that the shortening observed after colonisation was associated with increased cell turnover. They hypothesise that commensal bacteria contribute to enterocyte turnover through induction of inflammatory responses and cell apoptosis. In addition to the increased microvilli length, germ-free cod showed less microvilli per µm^2^ in the midgut than conventional reared larvae. Microvilli not only increase the cellular surface area for absorption of nutrients, they also increase the number of digestive enzymes present on the cell surface. Thus, the lower density of microvilli in the germ-free larvae could explain the apparent reduced nutrient uptake in the larvae. The distorted mitochondria observed in the germ-free cod larvae (Figure 6) also support the hypothesis of a physiological starvation state in these larvae. Hailey et al. [54] demonstrated how lipids from the mitochondrial membrane are utilised in the biogenesis of autophagosomes under starvation conditions.

The gene expression analysis indicates that certain elements of the innate immune system of cod larvae are “turned on” in the larval stage of the fish but are subsequently regulated by host–microbiota interactions. Solbakken et al. [22] described how infection by a pathogenic bacterium, *Francisella noatunensis*, dampens the intracellular immune response to allow intracellular persistence of the pathogen. Our results indicate that generation of reactive oxygen species (ROS) was lower in conventional and gnotobiotic larvae than in germ-free larvae. ROS generation is tightly linked to mitochondrial metabolism, and thus could be elevated in the disintegrating mitochondria, as observed by electron microscopy analysis (Figure 6). ROS is also produced during recognition of non-self-substances and the immune response processes [55]. The main enriched GO terms in germ-free larvae were related to defence responses and responses to reactive oxygen species. This presumably higher ROS activity despite no bacteria present might be a consequence of the unique immune system of cod, or because phagocytes of germ-free larvae are activated by components of the live feed (such as algae). During infection by *F. noatunensis,* downregulation of ROS production in phagosomes is hypothesised to contribute to this pathogen’s survival [22]. Another indication of high ROS generation in the germ-free cod was high expression of *immunoresponsive gene 1* (*irg1*). Irg1 controls macrophage function, by regulating metabolic pathways leading to increased mitochondrial ROS production that aids bacterial killing. In zebrafish, this gene was upregulated by bacterial infection [56], whereas in our experiment, the expression was lower in the fish exposed to bacteria.

Transcripts for key proteins involved in recognition of both peptidoglycans (PGLYRP) and lectins were also downregulated by the bacteria present in this study. Pglyrp proteins have been found to be expressed in zebrafish eggs, developing embryos and adult tissues that are in contact with the environment, and there are indications that they have an important role in the defence against bacteria in young fish [57,58]. Both rbl (Rhamnose-binding lectin) and fel (fish egg lectin) are known to enhance phagocytosis, and the expression of the genes is normally upregulated when the host is exposed to potentially harmful bacteria [59,60]. However, Thongda et al. [59] point to studies in catfish where *rbl* is highly upregulated by short-term fasting, indicating a link between the feeding status and the immune function. Thus, the downregulation of *rbl* in the gnotobiotic and conventional cod might be coupled to the observed transcriptional responses to fasting. Thus, enforcing the indications that the germ-free fish do not digest their food as well as the gnotobiotic and conventional fish.

It has previously been shown that the bacteria used in the gnotobiotic treatment are non-detrimental and may improve survival of cod larvae [25]. Thus, the downregulation of immune responses due to these bacteria may be a way of inducing tolerance to the colonising microbiota. However, similar downregulation observed in conventional cod larvae, where the microbiota was shown to have a slight but significantly negative effect on the survival, suggests that the immune system of the larvae is not capable of distinguishing friend from foe at this early life stage.

Several of the immune-related genes found to be downregulated in this experiment are involved in processes that have been reported to be upregulated by the presence of bacteria in gnotobiotic zebrafish and stickleback [16,61,62]. This illustrates how early host–microbiota responses differ across teleost species. In contrast to stickleback and zebrafish, Atlantic cod are heavily reliant on the innate immune response due to the loss of MHC-2, which is critical for initiation of antigen-specific immune response. In addition, cod toll-like receptor families (TLRs) have undergone genetic deletions and subsequent diversifications, possibly to compensate for the lack of the classical adaptive immunity [24,63]. This means that the type of receptors and downstream immune pathways will differ to some extent between cod and other vertebrates such as zebrafish and mice [64]. As an example, whereas LPS in high doses is lethal to mammals and zebrafish [61], Atlantic cod has a lower LPS response, with much higher LD50 values [64,65]. Analysis of the full genome revealed that Atlantic cod lacks TLR4, the mammalian LPS receptor that has a functional ortholog in zebrafish [21,66]. Despite the lack of a TLR4 ortholog, cod head kidney cells still reacted to LPS exposure by upregulation of certain immune and xenobiotic pathways [66].

### 4.3. Presence of Bateria Induces Host Responses Related to Inflammatory Responses and Signalling

Even though several genes related to the innate immune system seem to be downregulated by the presence of bacteria, other innate immune system responses were induced by the presence of bacteria. *Cxcl8* (*interleukin 8*) and *lect2* (*leukocyte-cell derived chemotaxin 2*) (upregulated in both 13 and 16 dph larvae) were upregulated in both conventional and gnotobiotic larvae (*cxcl8* just below the cut-off value of log2 0.8-fold change in gnotobiotic larvae). These genes that encode chemokines that attract neutrophils by chemotactic activity are induced by inflammatory stimuli caused by, e.g., microbial stress, and have been identified in teleosts earlier [67,68,69,70]. Both genes were also upregulated by in vitro LPS stimulation of cod cell cultures [66]. In fish, *lect2* is assumed to have an important role in the inflammatory response, promoting phagocytic activity of macrophages [71,72]. Since *lect2* was upregulated at both 13 and 16 dph, we suspect that this gene may be an early and important bacterial response in cod.

The KEGG pathway “bacterial invasion of epithelial cells” and GO terms related to signalling and signal transduction were enriched in conventional larvae compared with both gnotobiotic and germ-free larvae. This seems plausible, as compared to a gnotobiotic community consisting of only two probiotic candidates, the microbiota in the conventional treatment could give a higher invasion pressure, more host–microbe interactions due to higher species richness and thus more signalling and host responses. For example, a gene linked to the innate immune system, *irak1* (*interleukin-1 receptor-associated kinase 1*), was upregulated in conventional larvae compared with germ-free larvae. This gene plays a critical role in initiating the innate immune response against pathogens and has been shown to be upregulated in fish after pathogen challenge [73]. This implies that *irak1* might participate in antibacterial immunity. In gnotobiotic fish, the expression of *irak1* was at the same level as in germ-free fish, indicating that the probiotic candidates used in the gnotobiotic treatment were detected as non-pathogens by the fish.

*Slc2a11* was the most upregulated gene (log2 fold change of 2.3) in conventional compared with germ-free larvae, and it was also upregulated in conventional compared with gnotobiotic larvae. This gene encodes a glucose transporter protein GLUT11, belonging to class II of these proteins [74]. In fish, glucose transport is important for several reasons: blood glucose levels change rapidly in response to environmental disturbances, increased plasma glucose levels may be an indicator of stress and glucose intolerance has been documented in fish [75]. The expression of another GLUT protein, GLUT4, belonging to class I of these proteins and encoded by the *slc2a4* gene, was also upregulated in conventional fish compared to the germ-free fish. This is the only insulin-sensitive member of class I, and it is expressed in insulin-sensitive tissues, such as heart, muscle and adipose tissue in cod [76]. These findings indicate that the microbial community in the conventional rearing bottles induced more stress on the fish than in the gnotobiotic treatment. This is in line with the fact that potential detrimental bacteria were found in the conventional rearing bottles, and that the bacterial strains added to the gnotobiotic flasks had been characterised as probiotic candidates in an earlier study [25].

## 5. Conclusions

To conclude, our results indicate that bacteria actively downregulate certain cod larvae immune responses, facilitating bacterial colonisation of mucosal surfaces. This concept of “downregulation” contrasts previous findings in zebrafish and stickleback and emphasizes the role of evolutionary history, highlighting the need to study host–microbe interactions in several teleost species. Similar to what has been shown in studies with other vertebrates, bacterial colonisation improved the nutritional state of the cod larvae, evident at both transcriptional and micromorphological levels and materialised as differences in growth rate. This study illustrates the dynamics between water- and host-associated microbiota and increases our insight into how Atlantic cod larvae respond physiologically and transcriptionally to bacterial colonisation.

## Figures and Tables

**Table 1:** Average length, diameter and abundance of microvilli in germ-free and conventional cod larvae at 16 dph. *n* = 3 cod larvae per treatment were analysed.

Treatment									
	Microvillus Length (nm)			Microvillus Diameter (nm)			Abundance of Microvilli (µm^−2^)		
	Mean ± SE	Min	Max	Mean ± SE	Min	Max	Mean ± SE	Min	Max
Germ-free	2021.40 ± 31.62	1038.08	3183.59	107.01 ± 0.89	81.77	148.49	42.76 ± 1.03	25.39	68.43
Conventional	1703.04 ± 11.18	1211.07	2360.96	99.10 ± 0.53	77.51	123.92	54.14 ± 1.05	35.56	69.29

## Data Availability

Sequencing data are stored at European Nucleotide Archive, accession numbers ERS8484975-ERS8484994.

## Supplementary Materials

The following are available online at https://www.mdpi.com/article/10.3390/microorganisms10010024/s1, Figure S1: Kaplan–Meier survival curves, Figure S2: Dry weight of cod larvae, Figure S3: DGGE gel, Figure S4: Venn diagram, Table S1: List of down-regulated genes (cut-off value of log2 fold change > 0.8) in conventional cod larvae compared with germ-free cod larvae at 16 dph. Table S2: List of up-regulated genes (cut-off value of log2 fold change > 0.8) in conventional cod larvae compared with germ-free cod larvae at 16 dph. Table S3: List of down-regulated genes (cut-off value of log2 fold change > 0.8) in gnotobiotic cod larvae compared with germ-free cod larvae at 16 dph. Table S4: Enriched GO terms in conventional larvae compared with both germ-free and gnotobiotic cod larvae. Table S5: Taxonomy and most similar “type strains” for the six OTUs classified as Vibrio as inferred from the Ribosomal Database Project (RDP). Text S1: Verification of germ-free conditions, Text S2: Live feed and bacterial cultures, Text S3: Sampling procedures, Text S4: Statistical analyses.
