# Supplementary material for: Commensal and Opportunistic Bacteria Present in the Microbiota in Atlantic Cod (Gadus morhua) Larvae Differentially Alter the Hosts’ Innate Immune Responses
Source: Microorganisms. 2021 Dec 24;10(1):24. doi: 10.3390/microorganisms10010024 (PMC8779962; doi:10.3390/microorganisms10010024)
Supplement: Supplementary file 1 [file microorganisms-10-00024-s001.zip › Supplementary materials Figures S1 to S4.pdf]

Supplementary material

Commensal and opportunistic bacteria present in the microbiota in Atlantic cod (*Gadus morhua*) larvae differentially alter the hosts' innate immune responses.

Ragnhild Inderberg Vestrum[1$], Torunn Forberg[1$], Birgit Luef[1], Ingrid Bakke[1], Per Winge[2], Yngvar Olsen[2] and Olav Vadstein[1,*]
*Authors contributed equally

Affiliations
1. Department of Biotechnology and Food Science, Norwegian University of Science and Technology, Trondheim
2. Department of Biology, Norwegian University of Science and Technology, Trondheim

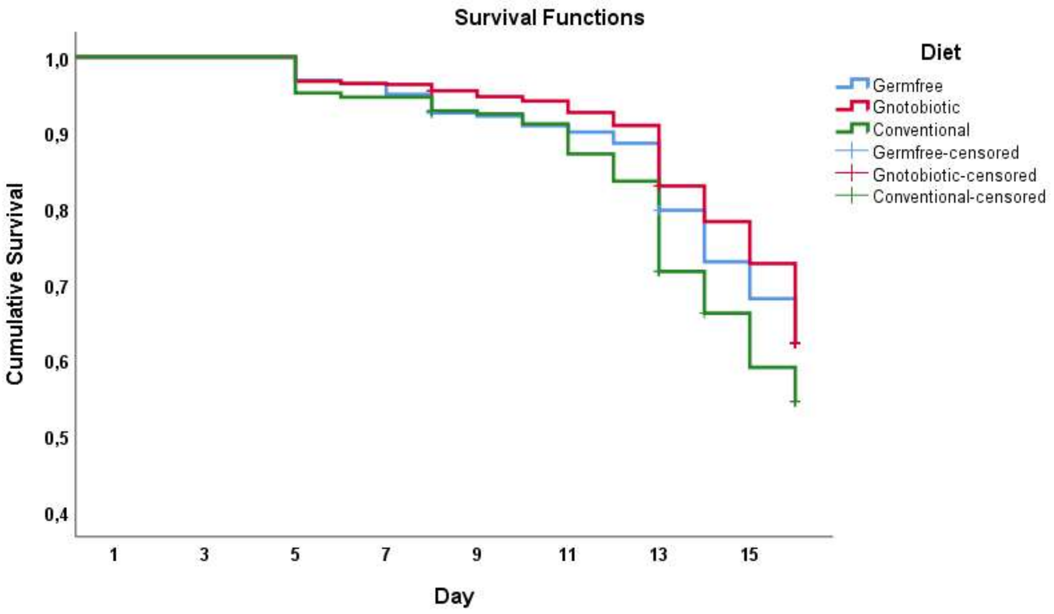

Figure S1: Kaplan-Meier survival curves. Censored events represent rearing flasks that were samples.

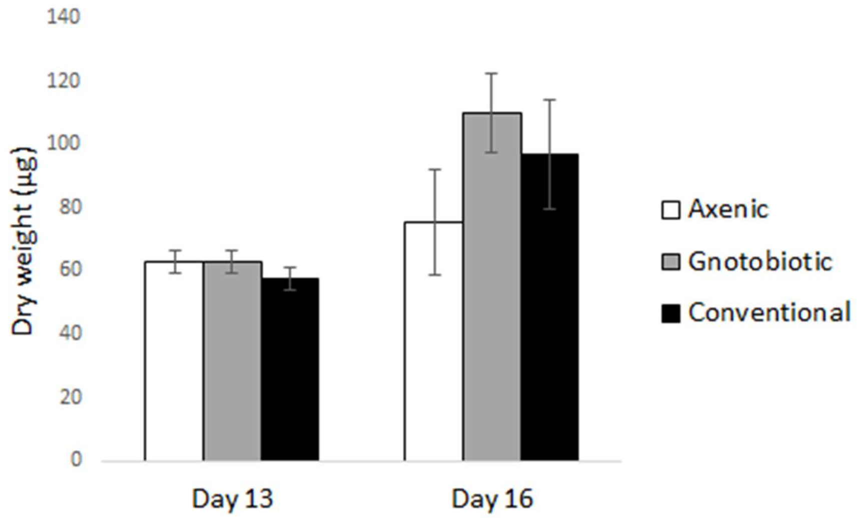

Figure S2: Dry weight of cod larvae. Dry weight (µg) of cod larvae in axenic (germ-free), gnotobiotic and conventional treatments at 13 and 16 dph.

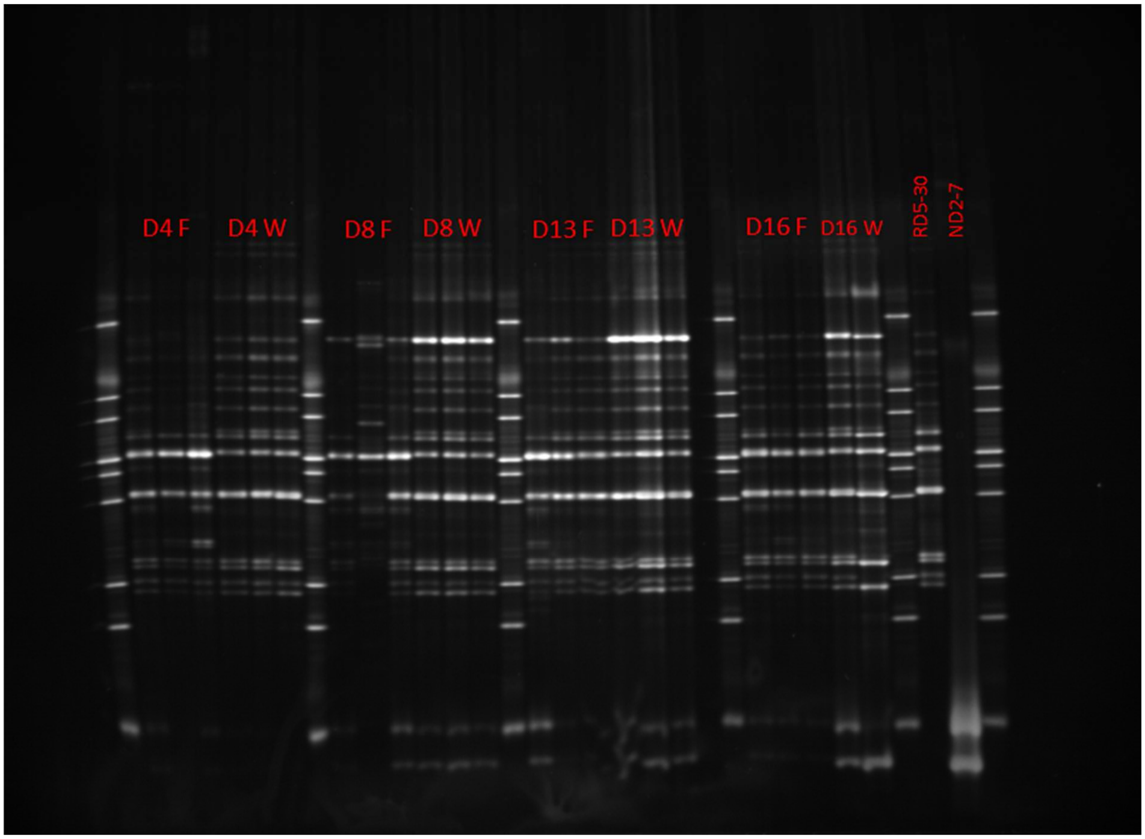

Figure S3: DGGE gel. Picture of DGGE gel with fish (F) and water (W) samples from the gnotobiotic treatment at 4, 8, 13 and 16 dph (D4-D16). Unmarked wells are ladders. Pure strains are in the two last wells; RD5-30 *Vibrio gallicus*., and ND2-7 *Microbacterium* sp.

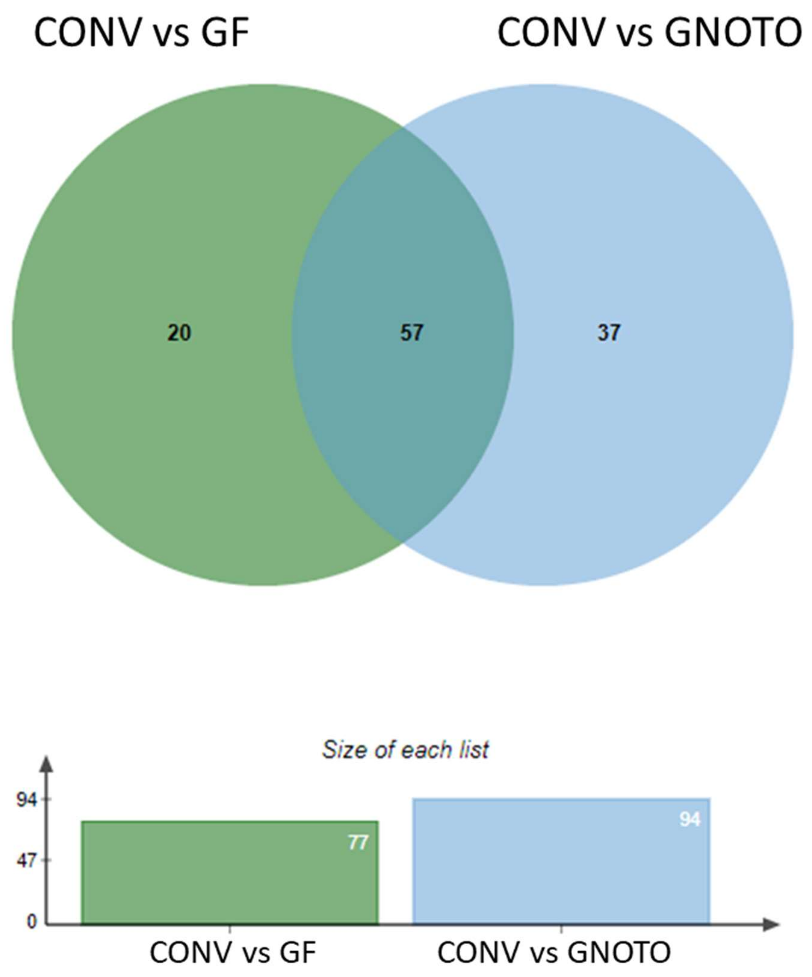

Figure S4: Venn diagram. Venn diagram showing the number of shared and unique genes up-regulated in conventional cod larvae compared with germ-free cod larvae (CONV vs GF), and up-regulated in conventional cod larvae compared with gnotnobiotic cod larvae (CONV vs GNOTO).
