# Supplementary material for: Commensal and Opportunistic Bacteria Present in the Microbiota in Atlantic Cod (Gadus morhua) Larvae Differentially Alter the Hosts’ Innate Immune Responses
Source: Microorganisms. 2021 Dec 24;10(1):24. doi: 10.3390/microorganisms10010024 (PMC8779962; doi:10.3390/microorganisms10010024)
Supplement: Supplementary file 1 [file microorganisms-10-00024-s001.zip › Supplementary materials Text S1 to S4.pdf]

Text S1: Verification of germ-free conditions

To verify germ-free conditions, samples of rearing water and live-feed cultures were routinely stained with SYBR green (SYBR Green I, Molecular Probes) before detection and quantification of DNA containing particles by flow cytometry (BD Accuri TM C6). Filtered autoclaved seawater was used as a reference to quantify the number of background particles. Water samples were also added to agar plates (M-65) to detect culturable contamination. Agar plates were incubated at 12 °C for up to one month. These methods verified that the germ-free bottles were germ-free.

Text S2: Live feed and bacterial cultures

Axenic *Isochrysis sp.* (CCAP 927/14) was purchased from "Culture collection of algae and protozoa" (http://www.ccap.ac.uk/index.htm) and used as feed for rotifers. Bacteria-free rotifers (*Brachionus* "Nevada") were obtained as described by Forberg et al. Rotifer and algae cultures were grown and kept as described by Forberg et al. Bacterial cultures were grown in M-65 broth (0.5 g peptone, 0.5 g tryptone and 0.5 g yeast extract in 800 mL FASW and 200 mL MilliQ water) at 20°C, harvested by centrifugation and resuspended in FASW before addition to rearing bottles. The density of bacteria was determined by measuring optical density (OD; absorbance at 660 nm) and conversion to cells/mL by strain specific conversion factors, using the measured OD in the following equations.

Vibrio gallicus (RD5-30) cells/mL = $2 \cdot 10^9$ OD – $5 \cdot 10^7$

Microbacterium sp. (ND2-7) cells/mL = $2 \cdot 10^9$ OD – $4 \cdot 10^8$

Text S3:

Cod larvae were collected with the rear end of a Pasteur pipette and spotted on a 1.5× 1.5 cm2 plankton net (100 µm) placed on a piece of tissue towel to drain off excess seawater. To ensure enough biological material, 5 larvae from each sampled bottle were pooled for DNA extraction, and 20, 15 and 10 larvae from each sampled bottle at 8, 13 and 16 dph, respectively, were pooled for RNA extraction. The net was immediately put into a SafeSeal micro tube (Sarstedt®) and then flash frozen in liquid nitrogen. Cod larvae were stored at −80 °C until further processing. Water was sampled at 1, 4, 8, 13 and 16 dph. The water was filtered through sterile 0.2 µm hollow fiber syringe filters (DynaGard, Microgon Inc., California), and stored at −20°C until further processing.

DNA and RNA extraction

DNA was extracted by use of the DNeasy Blood and Tissue Kit (Qiagen) according to the manufacturer's protocol. Total RNA was extracted by use of RNeasy mini kit® (Qiagen). Immediately prior to RNA extraction the frozen sample was flushed off the plankton net into a micro tube with 600 µL RLT buffer (RNeasy Lysis Buffer, Qiagen) with 1 % β-mercaptoethanol (ME) added. The samples were homogenized for 4 min at 25 Hz in a TissueLyzer® (Qiagen) using 5 mm stainless steel beads. Samples were treated according to the supplier's protocol "Purification of Total RNA fromAnimal Cells using Spin Technology" including the optional "on-column DNase treatment" as described in the kit manual. RNA was eluted in 50 µL RNase-free water, and RNasin® (Promega) was added to a final concentration of 1 U/µL. The RNA was quantified by measuring the absorbance at 260 nm, using a NanoDrop® ND-1000 spectrophotometer (NanoDrop technologies, Wilmington, DE, USA). The RNA integrity of samples was analysed by Agilent RNA 6000 Nanochip© and Agilent Bioanalyzer© (Agilent Technologies), showing RIN (RNA Integrity Number) values >8.

Text S4: Statistical analyses

Student’s t-test (unpaired) was used to investigate significance in differences in Shannon indices, abundance of individual DGGE bands and larval growth measurements. Ordination by Principal Coordinate Analysis (PCoA) based on Bray-Curtis similarities (1) was used to visualize differences between sample groups, and one-way and two-way PERMANOVA (2) based on Bray-Curtis similarities were used to test for statistically significant differences between sample groups. Similarity Percentage analysis (SIMPER) was used to identify OTUs responsible for differences (measured as Bray-Curtis similarities) between different sample groups. The multivariate analyses were performed using the program package PAST version 3.22 (3). Venn diagrams were created using jvenn (4). The Usearch commands Alpha_div and Sintax_summary, were used to calculate alpha diversity indices and to generate taxa summary tables (at various taxonomic levels as specified with the results), respectively. Data from the intestinal morphometric study were statistically analysed with IBM SPSS Statistics (SPSS for Windows, version 26.0; SPSS Inc., Chicago, IL, USA). A Welch-test was performed to investigate significantly differences in Microvilli length and abundance of microvilli μm^{-2}. Differences were considered statistically significant when $p \leq 0.05$.

1. Bray JR, Curtis JT. 1957. An ordination of the upland forest communities of southern Wisconsin. Ecological monographs 27:325-349.
2. Anderson MJ. 2001. A new method for non-parametric multivariate analysis of variance. Austral ecology 26:32-46.
3. Hammer Ø, Harper D, Ryan P. 2001. PAST-palaeontological statistics, ver. 1.89. Palaeontologia electronica 4.
4. Bardou P, Mariette J, Escudié F, Djemiel C, Klopp C. 2014. jvenn: an interactive Venn diagram viewer. BMC Bioinformatics 15:1-7.
