# Supplementary material for: Commensal and Opportunistic Bacteria Present in the Microbiota in Atlantic Cod (Gadus morhua) Larvae Differentially Alter the Hosts’ Innate Immune Responses
Source: Microorganisms. 2021 Dec 24;10(1):24. doi: 10.3390/microorganisms10010024 (PMC8779962; doi:10.3390/microorganisms10010024)
Supplement: Supplementary file 1 [file microorganisms-10-00024-s001.zip › Supplementary Table S1.pdf]

Table S1: List of down-regulated genes (cut-off value of log2 fold change > 0.8) in conventional cod larvae compared with germ-free cod larvae at 16 dph.

| **Accession number** | **Gene name** | **Description** | **Log2 fold change** | **Adjusted p-value** |
|---|---|---|---|---|
| ENSGAUG00000005408 | CUZD1 | Zona pellucida-like domain-containing protein | -2.792 | 0.000100535 |
| ENSGAUG00000012193 | EPX | Eosinophil peroxidase peroxidase-like | -2.407 | 5.89E-05 |
| ENSGAUG00000006436 | EPX | Eosinophil peroxidase | -1.784 | 0.010355002 |
| ENSGAUG00000015302 | TMPRSS9 | Transmembrane protease serine 9-like; partial ORF | -1.644 | 4.05E-06 |
| ENSGAUG00000015614_3 | C1QL3 | Complement C1q-like protein 3-like | -1.633 | 0.0008 |
| ENSGAUG00000017013 | MYOC | Myocilin | -1.555 | 0.00324 |
| ENSGAUG00000010274 | MPO | Myeloperoxidase precursor | -1.499 | 0.000508003 |
| ENSGAUG00000000611 | RBL | Rhamnose-binding lectin | -1.475 | 4.98E-07 |
| ENSGAUG00000004081 | HCE | low choriolytic enzyme-like, predicted | -1.457 | 4.42E-05 |
| ENSGAUG00000003520 | SEPP1 | Selenoprotein P | -1.451 | 0.000825 |
| ENSGAUG00000009502 | ctrb1 | Uncharacterized protein | -1.256 | 0.01677 |
| ENSGAUG00000005121 | CYBB | Cytochrome b-245 heavy chain-like | -1.251 | 3.92E-05 |
| ENSGAUG00000009693 | PRSS27 | Serine protease 27-like, predicted | -1.251 | 2.31E-05 |
| ENSGAUG00000012542 | APOD | Apolipoprotein D-like, predicted | -1.249 | 0.021925 |
| ENSGAUG00000012822 | ABCA12 | ATP-binding cassette sub-family A member 12-like, predicted | -1.219 | 0.001 |
| ENSGAUG00000001749_1 | GPRC6A | G-protein coupled receptor family C group 6 member A | -1.205 | 4.92E-07 |
| ENSGAUG00000011292 | SPINT2 | Kunitz-type protease inhibitor 2 precursor | -1.187 | 4.66E-05 |
| ENSGAUG00000001749 | GPRC6A | G-protein coupled receptor family C group 6 member A | -1.185 | 4.88E-07 |
| ENSGAUG00000020348 | FEL | Fish-egg lectin | -1.142 | 6.62E-05 |
| ENSGAUG00000000775 | Rhcg | Ammonium transporter Rh type C | -1.134 | 0.001845 |
| ENSGAUG00000014136 | PDLIM5 | PDZ and LIM domain protein 5-like | -1.089 | 0.000235 |
| ENSGAUG00000009769 | PGLYRP1 | N-acetylmuramoyl-L-alanine amidase-like | -1.065 | 0.000195495 |
| ENSGAUG00000008126 | ZGP16 | Zymogen granule membrane protein 16 | -1.061 | 0.00044 |
| ENSGAUG00000010286 | epx | Eosinophil peroxidase | -1.052 | 0.01751 |
| ENSGAUG00000009511_1 | ctrb2 | Chymotrypsin B | -1.047 | 0.02769 |

| | | | | |
|---|---|---|---|---|
| ENSGAUG00000015892 | ARHGAP6 | Rho GTPase-activating protein 6 | -1.041 | 7.34E-06 |
| ENSGAUG00000013172 | CeLA1 | Elastase-like protein | -1.023 | 0.03216 |
| ENSGAUG00000000080 | CTRL | Chymotrypsin-like protease CTRL-1 precursor | -1.022 | 0.02384 |
| ENSGAUG00000019115 | PLD3 | Phospholipase D3 | -1 | 0.000113925 |
| ENSGAUG00000003430 | Cpa2 | Carboxypeptidase A2 precursor | -0.99 | 0.02862 |
| ENSGAUG00000015335 | IGF1R | Insulin-like growth factor 1 receptor | -0.97 | 0.00041 |
| ENSGAUG00000006000 | Irg1 | Immune-responsive gene 1 protein-like | -0.966 | 0.000154035 |
| ENSGAUG00000006754 | MOV10 | Putative helicase mov-10-B.2-like, predicted | -0.961 | 0.01108 |
| ENSGAUG00000000958 | RNF213 | RING finger protein 213-like, predicted | -0.959 | 0.009085 |
| ENSGAUG00000009283 | NME2 | Nucleoside diphosphate kinase B isoform a | -0.946 | 0.015995 |
| ENSGAUG00000007716 | ZBTB4 | Uncharacterized protein | -0.945 | 0.017470097 |
| ENSGAUG00000008762 | predicted | Uncharacterized protein | -0.936 | 5.69E-05 |
| ENSGAUG00000017352 | bco2 | Beta,beta-carotene 9',10'-oxygenase-like, predicted | -0.935 | 0.007315 |
| FF406537.1 | SPINK2 | Serine protease inhibitor Kazal-type 2-like, predicted | -0.929 | 0.00178 |
| ENSGAUG00000016860 | DSP | Desmoplakin, predicted | -0.913 | 0.0199 |
| ENSGAUG00000005570 | Rbp3 | Retinol-binding protein 3-like | -0.913 | 0.00415 |
| ENSGAUG00000017471 | MSLN | Uncharacterized protein | -0.912 | 0.000955 |
| ENSGAUG00000008774 | OTOP2 | Otopetrin-2-like | -0.909 | 1.75E-05 |
| ENSGAUG00000008734 | UBL1 | Ubiquitin-like protein precursor | -0.902 | 0.02574 |
| ENSGAUG00000001451 | TRAF3IP2 | Adapter protein CIKS-like, predicted | -0.901 | 0.00058 |
| ENSGAUG00000007277 | CRYBB1 | Beta-crystallin B1-like, predicted | -0.901 | 0.00142 |
| ENSGAUG00000002315 | SDS | L-serine dehydratase/L-threonine deaminase-like, predicted | -0.891 | 0.01677 |
| EX725973.1 | SPINK1 | Pancreatic secretory trypsin inhibitor | -0.889 | 0.00548 |
| ENSGAUG00000014401 | Cfad | Counting factor associated protein D-like, predicted | -0.885 | 0.002775 |
| ENSGAUG00000012105 | MYOC | Myocilin-like, predicted | -0.885 | 0.00214 |
| ENSGAUG00000005518 | NKX3-1 | Homeobox protein Nkx-3.1-like | -0.882 | 0.00166 |
| ENSGAUG00000014896 | ApoL1 | ApoL super family protein | -0.879 | 0.00037 |
| ENSGAUG00000010299 | CMPK2 | UMP-CMP kinase 2, mitochondrial-like, predicted | -0.879 | 0.00459 |
| ENSGAUG00000005387 | predicted | Uncharacterized protein | -0.876 | 0.000365 |
| ENSGAUG00000019464 | LRRC16A | leucine-rich repeat-containing protein 16A-like, predicted | -0.863 | 0.010275 |

| | | | | |
|---|---|---|---|---|
| ENSGAUG00000013953 | RSRC2 | Arginine/serine-rich coiled-coil protein 2-like, predicted | -0.86 | 0.015415 |
| ENSGAUG00000011643 | CFAP74 | Uncharacterized protein KIAA1751 homolog, predicted | -0.857 | 0.0139 |
| ENSGAUG00000012948_1 | PRSS1 | Trypsin-1 precursor | -0.853 | 0.04893 |
| ENSGAUG00000016090 | GTPBP4 | Nucleolar GTP-binding protein 1, predicted | -0.851 | 0.004785 |
| ENSGAUG00000007955_3 | PNLIPRP1 | Pancreatic lipase-related protein | -0.843 | 0.033135 |
| ENSGAUG00000016793 | POR | NADPH--cytochrome P450 reductase-like, predicted | -0.842 | 0.011765 |
| ENSGAUG00000016899 | NID2 | Nidogen-2, predicted | -0.842 | 0.000645 |
| ENSGAUG00000019687 | DVL1 | Segment polarity protein dishevelled homolog DVL-1-like isoform 2, predicted | -0.841 | 0.00225781 |
| ENSGAUG00000004250 | col6a1 | Collagen alpha-1(VI) chain-like, predicted | -0.84 | 5.55E-05 |
| ENSGAUG00000006831 | CRYBA1 | Beta-crystallin A1-like, predicted | -0.835 | 0.001375225 |
| ENSGAUG00000009144 | SErPINA10 | Protein Z-dependent protease inhibitor-like, predicted | -0.835 | 0.000538925 |
| ENSGAUG00000000514 | SQSTM1 | Sequestosome-1 | -0.833 | 0.042225 |
| ENSGAUG00000013176 | CAPN3 | Calpain-3-like, predicted | -0.828 | 0.000385 |
| ENSGAUG00000018097 | Bcl6 | B-cell lymphoma 6 protein homolog, predicted | -0.826 | 0.00096 |
| ENSGAUG00000000178 | atp6v1b | V-type proton ATPase subunit B, brain isoform-like, predicted | -0.823 | 0.000495 |
| ENSGAUG00000018340 | CAPN2 | Calpain-2 catalytic subunit | -0.821 | 0.021775 |
| ENSGAUG00000003005 | CKB | Creatine kinase B-type | -0.82 | 0.0011 |
| ENSGAUG00000000260 | anxa11 | Annexin A11b | -0.818 | 0.00041 |
| ENSGAUG00000004348 | PPP3CC | Serine/threonine-protein phosphatase 2B catalytic subunit gamma isoform, predicted | -0.817 | 2.13E-05 |
| ENSGAUG00000009193 | and1 | Actinodin1 precursor | -0.816 | 0.00972 |
| ENSGAUG00000019589 | crybb1 | Beta-crystallin B1-like, predicted | -0.814 | 0.000765 |
| ENSGAUG00000002737 | IRF4 | Interferon regulatory factor 4-like | -0.81 | 0.01243 |
| ENSGAUG00000008523 | predicted | Zona pellucida-like domain-containing protein | -0.809 | 0.002275 |
| ENSGAUG00000000027 | COL24A1 | Collagen alpha-1(X) chain-like isoform 2, predicted | -0.802 | 0.02197 |
