# Supplementary material for: Commensal and Opportunistic Bacteria Present in the Microbiota in Atlantic Cod (Gadus morhua) Larvae Differentially Alter the Hosts’ Innate Immune Responses
Source: Microorganisms. 2021 Dec 24;10(1):24. doi: 10.3390/microorganisms10010024 (PMC8779962; doi:10.3390/microorganisms10010024)
Supplement: Supplementary file 1 [file microorganisms-10-00024-s001.zip › Supplementary Table S2.pdf]

Table S2: List of up-regulated genes (cut-off value of log2 fold change > 0.8) in conventional cod larvae compared with germ-free cod larvae at 16 dph.

Accession number	Gene name	Description	Log2 fold change	Adjusted p-value
ENSGAUG00000001043	SLC2A11	Solute carrier family 2 (facilitated glucose transporter), member 11-like	2.256	0.001395
ENSGAUG00000000097	LECT2	Leukocyte cell-derived chemotaxin 2	2.104	0.00261
ENSGAUG00000016719	CXCL8	Interleukin 8; partial ORF	1.898	0.018435
ENSGAUG00000009351	PRAF2	PRA1 family protein 2	1.779	0.02513
ENSGAUG00000014818	EMR1	EGF-like module-containing mucin-like hormone receptor-like 1 precursor	1.401	0.000755
ENSGAUG00000016286	PKD2	Polycystin-2-like protein	1.368	0.00226
ENSGAUG00000019259	B3GALTL	Beta-1,3-glucosyltransferase-like, predicted	1.283	0.000725
ENSGAUG00000018326	NKX1-2	NK1 transcription factor-related protein 2	1.246	0.001345
ENSGAUG00000007503	AKAP12	Uncharacterized protein	1.231	0.00017
ENSGAUG00000012893	ZNF423	Zinc finger protein 423-like	1.227	0.00277
ENSGAUG00000002110	RNF38	Uncharacterized protein	1.221	0.001335
ENSGAUG00000017530	KBTBD4	Kelch repeat and BTB domain-containing protein 4, predicted	1.213	0.00369
ENSGAUG00000004548	CIR1	Corepressor interacting with RBPJ 1-like, predicted	1.201	3.34E-06
ENSGAUG00000010641	PIK3CA	Phosphatidylinositol-4,5-bisphosphate 3-kinase catalytic subunit alpha isoform-like, predicted	1.191	0.00599
ENSGAUG00000013093	ENTHD2	Uncharacterized protein C17orf56 homolog, predicted	1.19	0.01004
ENSGAUG00000018999	NDNF	Fibronectin type-III domain-containing protein C4orf31 homolog, predicted	1.181	6.54E-05
ENSGAUG00000013599	CHD3	Chromodomain-helicase-DNA-binding protein 3, predicted	1.176	0.0036
ENSGAUG00000011988	H2B	Histone H2B 1/2-like, predicted	1.17	0.00229
ENSGAUG00000017429	LPPR5	Lipid phosphate phosphatase-related protein type 5-like, predicted	1.164	0.01714
ENSGAUG00000010222_2	Mboat2	Membrane-bound O-acyltransferase domain-containing protein 2-like, predicted	1.142	0.00038
JK781143.1	COX17	Cytochrome c oxidase copper chaperone	1.138	0.00068
ENSGAUG00000006082	CHST12	Carbohydrate sulfotransferase 12-like, predicted	1.132	0.00066

ENSGAUG00000017772	DNMBP	Dynamin-binding protein-like, predicted	1.125	0.001945
ENSGAUG00000000279	KANSL1L	Uncharacterized protein C2orf67 homolog, predicted	1.1	0.01597
JK759241.1	predicted	Uncharacterized protein	1.099	0.00071
ENSGAUG00000007979	ZNF532	Zinc finger protein 532-like, predicted	1.083	0.015505
ENSGAUG00000001682	PLEKHG1	Pleckstrin homology domain-containing family G member 1-like, predicted	1.069	0.00248
ENSGAUG00000010186	RBM22	Pre-mRNA-splicing factor RBM22-like, predicted	1.063	0.001225
ENSGAUG00000005024	LNP1	Uncharacterized protein	1.047	0.00225
ENSGAUG00000002564	GUCY2C	Heat-stable enterotoxin receptor-like, predicted	1.029	0.006855
ENSGAUG00000009053	IRAK1	Interleukin-1 receptor activated kinase 1	1.027	0.000825
ENSGAUG00000009691	BBs9	PTHB1-like protein	1.017	0.00161
ENSGAUG00000019046_2	MAtr3	Uncharacterized protein	1.012	0.047975
ENSGAUG00000004448	TMC8	Transmembrane channel-like protein 7	0.993	0.001695
ENSGAUG00000006025	RFC3	Replication factor C subunit 3-like, predicted	0.981	0.012395
ATLCOD1ESTi24380	KMT5A	N-lysine methyltransferase SETD8-A-like, predicted	0.96	0.00123
ENSGAUG00000010843	NRIP2	Nuclear receptor-interacting protein 2-like, predicted	0.956	0.00219
ENSGAUG00000000191	DYRK4	Dual specificity tyrosine-phosphorylation-regulated kinase 4-like, predicted	0.952	0.002485
ENSGAUG00000006720	PDLIM7	PDZ and LIM domain protein 7	0.949	0.00219
ENSGAUG00000005277	ASPH	Aspartate beta-hydroxylase	0.947	6.33E-05
ENSGAUG00000016074	AAMP	Angio-associated migratory cell protein-like, predicted	0.938	0.002585
ENSGAUG00000006980_2	ASPM	Abnormal spindle-like microcephaly-associated protein homolog, predicted	0.937	0.002445
ENSGAUG00000002655	UBE2V2	Ubiquitin-conjugating enzyme E2 variant 2	0.937	0.01275
ATLCOD1ESTi11511	DIS3	Exosome complex exonuclease RRP44	0.936	0.000325
ENSGAUG00000007757	SLC2A4	Solute carrier family 2, facilitated glucose transporter member 4-like, predicted	0.935	0.002195
ENSGAUG00000003702	OSBPL2	Oxysterol-binding protein-related protein 2, predicted	0.925	0.00186
ENSGAUG00000003347	DNAJC21	DNAJ homolog subfamily C member 21-like, predicted	0.923	0.00274
ENSGAUG00000017967	RFESD	Rieske domain-containing protein	0.921	0.0017
ENSGAUG00000014477	SSPO	SCO-spondin, predicted	0.914	0.023125

ENSGAUG00000016130	THRA	Thyroid hormone receptor alpha, predicted	0.897	0.001265
ENSGAUG00000010653	STAC2	SH3 and cysteine-rich domain-containing protein-like, predicted	0.896	0.001435
ENSGAUG00000006651	BCAT5	Branched-chain-amino-acid aminotransferase, cytosolic	0.895	0.000355
ENSGAUG00000018119	MRGBP	MRG-binding protein-like isoform 2, predicted	0.892	0.00036281
ENSGAUG00000010089	predicted	Uncharacterized protein	0.891	0.009605
ENSGAUG00000012154	PRKRIR	Uncharacterized protein	0.886	0.000175
ENSGAUG00000016339	NDUFA4L2	NADH dehydrogenase [ubiquinone] 1 alpha subcomplex subunit 4-like 2-like, predicted	0.882	0.030095
ENSGAUG00000003179	LUC7L3	Uncharacterized protein	0.873	0.00017596
ENSGAUG00000020508	RAP2B	Ras-related protein Rap-2b-like, predicted	0.869	0.00092218
ENSGAUG00000013318	PHF5A	PHD finger-like domain-containing protein 5A	0.868	0.01581
FG324761.1	ARHGAP11A	Rho GTPase-activating protein 11A-like, predicted	0.863	0.00556
ATLCOD1ESTi27138	SMAP	Uncharacterized protein	0.859	0.00467
ENSGAUG00000006434	SH3RF1	E3 ubiquitin-protein ligase SH3RF1-like, predicted	0.858	0.013665
ATLCOD1ESTi10705	VCL	Vinculin-like, predicted	0.855	0.00064
ENSGAUG00000016680	GNRH3	Progonadoliberin-3 isoform 1, predicted	0.849	0.00504
ENSGAUG00000011627	ARHGAP44	Rho GTPase-activating protein 44-like, predicted	0.849	0.0163
ENSGAUG00000009106	HDHD5	Cat eye syndrome critical region protein 5-like, predicted	0.847	0.014685
ENSGAUG00000014465	DCUN1D1	DCN1-like protein 5	0.843	0.004535
ENSGAUG00000018394	ITGB1	Integrin, beta 1b.2 precursor	0.843	0.00396
ENSGAUG00000013372	COl11A2	Collagen alpha-2(IX) chain-like, predicted	0.838	0.00833
JK759682.1	DPY30	Protein dpy-30 homolog, predicted	0.838	0.00028
ENSGAUG00000000692	predicted	Uncharacterized protein	0.838	0.002955
JK775390.1	NSMCE2	E3 SUMO-protein ligase NSE2-like, predicted	0.837	0.00461
ENSGAUG00000015650	C1orf131	Uncharacterized protein C1orf131-like, predicted	0.831	0.00488
ENSGAUG00000019745	predicted	Uncharacterized protein	0.821	0.00644
ENSGAUG00000007235_1	HES4	Transcription factor HES-4-like, predicted	0.82	0.00453
ATLCOD1ESTi13247	CHTF8	Chromosome transmission fidelity protein 8 homolog, predicted	0.814	0.03279
ENSGAUG00000010222	Mboat2	Membrane-bound O-acyltransferase domain-containing protein 2-like, predicted	0.814	0.00071

ENSGAUG00000008020	GPC5	Uncharacterized protein	0.811	0.003625
ENSGAUG00000015858	FAT3	Protocadherin Fat 3-like, predicted	0.805	0.00518
ENSGAUG00000004374	HIST1H1A	Uncharacterized protein	0.804	0.00863
ENSGAUG00000006617	OTUD5	OTU domain-containing protein 5-A-like, predicted	0.803	0.00351
CUST_37315_PI425838995		Unknown probe; no match against cod transcripts or sequences in the cod genome	1.258	0.00206
CUST_37314_PI425838995		Unknown probe; no match against cod transcripts or sequences in the cod genome	1.224	0.00227
CUST_20633_PI425838995		Unknown probe; no match against cod transcripts or sequences in the cod genome	1.026	0.01041
CUST_17997_PI425838995		Unknown probe; no match against cod transcripts or sequences in the cod genome	0.978	0.0005
ES478198.1		Unknown protein; this transcript has no score against other known proteins	1.022	0.00059
JK758476.1		Unknown transcript; no similarity to other known proteins	1.28	0.0033
ENSGAUG00000000597		Unknown transcript; no similarity to other known proteins	1.266	0.00138
JK770651.1		Unknown transcript; no similarity to other known proteins	1.163	0.01395
ATLCOD1ESTi39954		Unknown transcript; no similarity to other known proteins	0.952	0.00043
JK759699.1		Unknown transcript; no similarity to other known proteins	0.89	0.0005
ENSGAUG00000010415		Unknown transcript; no similarity to other known proteins	0.853	0.00077
JK782837.1		Unknown transcript; no similarity to other known proteins	0.846	0.00223
JK758705.1		Unknown transcript; no similarity to other known proteins	0.845	0.04586
JK760351.1		Unknown transcript; no similarity to other known proteins	0.833	0.00728
JK764075.1		Unknown transcript; no similarity to other known proteins	0.82	0.00094
FG318028.1		Unknown transcript; no similarity to other known proteins	0.818	0.00083
