# Supplementary material for: Commensal and Opportunistic Bacteria Present in the Microbiota in Atlantic Cod (Gadus morhua) Larvae Differentially Alter the Hosts’ Innate Immune Responses
Source: Microorganisms. 2021 Dec 24;10(1):24. doi: 10.3390/microorganisms10010024 (PMC8779962; doi:10.3390/microorganisms10010024)
Supplement: Supplementary file 1 [file microorganisms-10-00024-s001.zip › Supplementary Table S3.pdf]

Table S3: List of down-regulated genes (cut-off value of log2 fold change > 0.8) in gnotobiotic cod larvae compared with germ-free cod larvae at 16 dph.

Accession	Gene name	Description	Log2 fold change	Adjusted p-value
ENSGAUG00000005408	CUZD1	Zona pellucida-like domain-containing protein	-2.668	0.000542
ENSGAUG00000006436	EPX	Eosinophil peroxidase	-1.688	0.011975
ENSGAUG00000012193	EPX	Eosinophil peroxidase	-1.65	0.01609
ENSGAUG00000000611	RBL	Rhamnose-binding lectin	-1.644	3.78E-07
ENSGAUG00000008126	ZGP16	Zymogen granule membrane protein 16	-1.509	3.22E-05
ENSGAUG00000014891	ZG16p	Zymogen granule membrane protein 16-like	-1.292	4.84E-05
ENSGAUG00000001749	GPRC6A	G-protein coupled receptor family C group 6 member A	-1.287	1.65E-07
ENSGAUG00000010274	MPO	Myeloperoxidase precursor	-1.253	0.014335
ENSGAUG00000005121	CYBB	Cytochrome b-245 heavy chain-like	-1.252	9.33E-05
ENSGAUG00000001749_1	GPRC6A	G-protein coupled receptor family C group 6 member A	-1.191	7.31E-07
ENSGAUG00000006000	Irg1	Immune-responsive gene 1 protein-like	-1.178	3.56E-05
ENSGAUG00000020348	FEL	Fish-egg lectin	-1.068	0.000455
ENSGAUG00000014896	ApoL1	ApoL super family protein	-0.985	0.000575
ENSGAUG00000015892	ARHGAP6	Rho GTPase-activating protein 6	-0.915	5.90E-05
ENSGAUG00000005518	NKX3-1	Homeobox protein Nkx-3.1-like	-0.909	0.018765
ENSGAUG00000007017	EHF	Ets homologous factor related protein	-0.894	0.00049
ENSGAUG00000005387	predicted	Uncharacterized protein	-0.891	0.002385
ENSGAUG00000009769	PGLYRP1	N-acetylmuramoyl-L-alanine amidase-like	-0.879	0.01099
ENSGAUG00000015302	TMPRSS9	Transmembrane protease serine 9-like	-0.85	0.01579
ENSGAUG00000006012	predicted	YrdC domain-containing protein	-0.819	0.017235
ENSGAUG00000009693	PRSS27	Serine protease 27-like, predicted	-0.817	0.013225
ATLCOD1ESTi20380		Unknown transcript; no similarity to other known proteins	-1.39	0.00072
JW189634.1		Unknown transcript; no similarity to other known proteins	-1.38	0.00305
