# Supplementary material for: Commensal and Opportunistic Bacteria Present in the Microbiota in Atlantic Cod (Gadus morhua) Larvae Differentially Alter the Hosts’ Innate Immune Responses
Source: Microorganisms. 2021 Dec 24;10(1):24. doi: 10.3390/microorganisms10010024 (PMC8779962; doi:10.3390/microorganisms10010024)
Supplement: Supplementary file 1 [file microorganisms-10-00024-s001.zip › Supplementary Table S4.pdf]

Table S4: Enriched GO terms in conventional larvae compared with both germ-free and gnotobiotic cod larvae.

GO Term	Gene Count	PValue	Genes
GO:1902531~regulation of intracellular signal transduction	12	0.005	IRAK1, SH3RF1, RFC3, PLEKHG1, ARHGAP44, PKD2, AKAP12, PIK3CA, ASPH, ITGB1, CHD3, DNMBP
GO:0043085~positive regulation of catalytic activity	11	0.008	IRAK1, DCUN1D1, RFC3, PLEKHG1, ARHGAP44, CHTF8, PKD2, PIK3CA, ASPH, ITGB1, DNMBP
GO:0035556~intracellular signal transduction	15	0.008	IRAK1, SH3RF1, AKAP12, GUCY2C, ITGB1, NDNF, DNMBP, RFC3, STAC2, PLEKHG1, ARHGAP44, PKD2, PIK3CA, ASPH, CHD3
GO:0031585~regulation of inositol 1,4,5-trisphosphate-sensitive calcium-release channel activity	2	0.01	PKD2, ASPH
GO:1900262~regulation of DNA-directed DNA polymerase activity	2	0.02	RFC3, CHTF8
GO:1900264~positive regulation of DNA-directed DNA polymerase activity	2	0.02	RFC3, CHTF8
GO:0050790~regulation of catalytic activity	13	0.02	IRAK1, SH3RF1, CHTF8, ITGB1, SSPO, DNMBP, RFC3, DCUN1D1, PLEKHG1, ARHGAP44, PKD2, PIK3CA, ASPH
GO:0044093~positive regulation of molecular function	11	0.02	IRAK1, DCUN1D1, RFC3, PLEKHG1, ARHGAP44, CHTF8, PKD2, PIK3CA, ASPH, ITGB1, DNMBP
GO:0009966~regulation of signal transduction	14	0.03	IRAK1, SH3RF1, THRA, AKAP12, ITGB1, DNMBP, RFC3, PLEKHG1, ARHGAP44, PKD2, PIK3CA, ASPH, CHD3, ZNF423
GO:0051347~positive regulation of transferase activity	6	0.03	IRAK1, DCUN1D1, RFC3, CHTF8, PKD2, PIK3CA
GO:0019219~regulation of nucleobase-containing compound metabolic process	18	0.03	RBM22, IRAK1, THRA, CHTF8, AKAP12, MRGBP, NRIP2, DPY30, RFC3, CIR1, NKX1-2, HES4, RNF38, PKD2, PIK3CA, ASPH, CHD3, ZNF423
GO:0048514~blood vessel morphogenesis	5	0.04	AAMP, PKD2, PIK3CA, ITGB1, NDNF
GO:0035023~regulation of Rho protein signal transduction	3	0.04	PLEKHG1, ITGB1, DNMBP
GO:2000811~negative regulation of anoikis	2	0.04	PIK3CA, ITGB1
GO:0051056~regulation of small GTPase mediated signal transduction	4	0.05	PLEKHG1, ARHGAP44, ITGB1, DNMBP
GO:0042994~cytoplasmic sequestering of transcription factor	2	0.05	THRA, PKD2
GO:0071456~cellular response to hypoxia	3	0.05	IRAK1, SLC2A4, NDNF
