# Supplementary material for: Commensal and Opportunistic Bacteria Present in the Microbiota in Atlantic Cod (Gadus morhua) Larvae Differentially Alter the Hosts’ Innate Immune Responses
Source: Microorganisms. 2021 Dec 24;10(1):24. doi: 10.3390/microorganisms10010024 (PMC8779962; doi:10.3390/microorganisms10010024)
Supplement: Supplementary file 1 [file microorganisms-10-00024-s001.zip › Supplementary Table S5.pdf]

Table S5: Taxonomy and most similar "type strains" for the six OTUs classified as *Vibrio* as inferred from the Ribosomal Database Project (RDP).

**OTU**	**Type strain (closest matches)**	**S_ab score***
OTU 829	*Vibrio lentus, Vibrio tasmaniensis, Vibrio kanaloae, Vibrio atlanticus, Vibrio hemicentroti*	0.945
OTU 2	*Vibrio lentus, Vibrio tasmaniensis, Vibrio kanaloae, Vibrio atlanticus, Vibrio hemicentroti*	1.000
OTU 233	*Vibrio lentus, Vibrio tasmaniensis, Vibrio kanaloae, Vibrio atlanticus, Vibrio hemicentroti*	0.884
OTU 11	*Vibrio ichthyoenteri, Vibrio neptunius, Vibrio brasiliensis, Vibrio xuii, Vibrio rotiferianus, Vibrio campbellii, Vibrio hepatarius, Vibrio sinaloensis, Vibrio sagamiensis, Vibrio alfacsensis*	1.000
OTU 61	*Vibrio areningrae*	1.000
OTU 402	*Vibrio anguillarum*	0.946

* A seqmatch score (S_ab) were reported between every closely related sequence, which represents the number of unique 7-base oligomers shared between the sample sequence and a given RDP sequence divided by the lowest number of unique oligos in either of the two sequences (Wang et al. 2007).
